# Angehörigenbetreuung auf Intensivstationen: Übersicht und Update

**DOI:** 10.1007/s00063-022-00915-7

**Published:** 2022-04-08

**Authors:** Magdalena Hoffmann, Peter Nydahl, Maria Brauchle, Christine Schwarz, Karin Amrein, Marie-Madlen Jeitziner

**Affiliations:** 1 Klinische Abteilung für Endokrinologie und Diabetologie, Universitätsklinik für Innere Medizin, Medizinische Universität Graz, Auenbruggerplatz 15, 8036 Graz, Österreich; 2 Research Unit for Safety and Sustainability in Health Care c/o Klinische Abteilung für plastische, ästhetische und rekonstruktive Chirurgie, Universitätsklinik für Chirurgie, Medizinische Universität Graz, Graz, Österreich; 3 Stabsstelle für Qualitäts- und Risikomanagement, LKH-Universitätsklinikum Graz, Graz, Österreich; 4 Pflegewissenschaft und -entwicklung, Universitätsklinikum Schleswig-Holstein, Kiel, Deutschland; 5 Abteilung für Anästhesie und Intensivmedizin, Landeskrankenhaus Feldkirch, Feldkirch, Österreich; 6 Universitätsklinik für Intensivmedizin, Universitätsspital Bern (Inselspital), Universität Bern, Bern, Schweiz; 7 Pflegewissenschaft – Nursing Science (INS), Universität Basel, Medizinische Fakultät, Basel, Schweiz; 8 Departement Public Health (DPH), Basel, Schweiz

**Keywords:** COVID-19, Pandemien, Familie, Psychische Belastung, Kommunikation, COVID-19, Pandemics, Family, Psychological stress, Communication

## Abstract

**Hintergrund:**

Angehörige von kritisch Kranken auf der Intensivstation („intensive care unit“, ICU) sind in einer herausfordernden Situation: Sie befinden sich häufig in einer existenziellen Krise mit einer großen emotionalen Belastung, gleichzeitig sind sie oftmals aktiv in therapeutische Entscheidungen mit eingebunden. Die Besuchsrestriktionen während der Pandemie aufgrund der Coronaviruserkrankung 2019 (COVID-19) haben viele Rahmenbedingungen für die Angehörigenbegleitung geändert und so die Betreuung von Angehörigen schwieriger gemacht.

**Ziel:**

Ziel der Publikation ist die Darstellung der aktuellen und neuen Entwicklungen in der Angehörigenbegleitung von kritisch Kranken auf Intensivstationen im Rahmen einer narrativen Übersichtsarbeit.

**Ergebnisse:**

In den letzten Jahren wurden zahlreiche Maßnahmen und Projekte zur Angehörigenbegleitung entwickelt, die sich den folgenden 6 Bereichen zuordnen lassen: 1) Anwesenheit der Angehörigen, 2) proaktive Einbindung in die Betreuung, 3) strukturierte Kommunikation und Information sowie Onlineangebote, 4) multidisziplinäre Zusammenarbeit, 5) Aufgaben der Organisationsleitung und 6) Follow-up-Angebote. Die Evidenz und der derzeitige Implementierungsstand der Maßnahmen sind international und national sehr heterogen.

**Schlussfolgerungen:**

Maßnahmen zur Angehörigenbetreuung sind vielfältig und können zum Teil auch unter Besuchsrestriktionen umgesetzt werden. Neuere Entwicklungen im digitalen Bereich ermöglichen zunehmend auch virtuelle Besuche und einen ergänzenden Informationsaustausch zwischen dem Team der ICU und den Angehörigen.

## Abstract (en)

**Background:**

Relatives of patients in the intensive care unit (ICU) face a challenging situation: they often experience an existential crisis with great emotional stress and at the same time they are often actively involved in therapeutic decisions. The visiting restrictions of the coronavirus disease 2019 (COVID-19) pandemic have created new challenges in providing support to relatives.

**Objectives:**

The aim of this work is to present current and new developments in supporting relatives of critically ill patients in the form of a narrative review.

**Results:**

In recent years, numerous new approaches and projects to support relatives have been developed. They can be assigned to the following six areas: 1) presence of relatives in the ICU, 2) proactive involvement in care, 3) structured communication/information and online offers, 4) multidisciplinary cooperation, 5) organizational management and 6) follow-up offers. The evidence and the current implementation status of these measures are very heterogeneous internationally and nationally.

**Conclusions:**

Measures for providing support for ICU relatives are diverse. Some can even be implemented despite visit bans. Recent digital developments enable virtual visits and a better exchange of information between the ICU team and relatives.

Die Angehörigenbegleitung auf der Intensivstation (Intensive Care Unit, ICU) ist eine wichtige und gleichzeitig herausfordernde Aufgabe für die Mitarbeiter*innen. Dabei haben sich die Rolle und die Einflussmöglichkeiten der Angehörigen in den letzten Jahren stetig verändert. Neben der Ausweitung der Besuchszeiten wurde auch die aktive Beteiligung der Angehörigen beispielsweise in pflegerischen Bereichen Schritt für Schritt vergrößert. Ebenso wird der Follow-up-Betreuung eine immer größere Bedeutung zugeschrieben und es findet sich dazu eine Vielzahl an unterschiedlichen Maßnahmen. Es folgt eine Übersicht an Maßnahmen mit Fokus auf digitale Möglichkeiten und Follow-up-Angebote in der Angehörigenbegleitung.

## Hintergrund

Zu den Angehörigen von Patient*innen auf der ICU zählen die Menschen, zu denen die Patient*innen eine aus ihrer Sicht bedeutsame Beziehung haben, wie Ehepartner*in, Kinder, Verwandte und enge Freund*innen [6]. Die Aufnahme von Patient*innen auf die ICU stürzt viele Angehörige in eine Lebenskrise. In dieser erleben die Angehörigen eine hohe emotionale und physische Belastung. Die große Angst, ob die*der Patient*in überhaupt überlebt, wie die Prognose ist oder wie das weitere gemeinsame Leben gestaltet werden kann, löst bei den Angehörigen große Unsicherheit aus. Angehörige leiden dabei häufig unter Stress, depressiven Gefühlen und Schlafstörungen [2, 7, 10, 18, 21, 22]. Schmidt und Azoulay sowie Rushinova et al. berichten, dass rund 70–78 % der Angehörigen von starker Angst betroffen sind. Daneben zeigen 35–54 % der Angehörigen auch depressive Symptome [28, 29]. Mitunter können die Belastungen so groß werden, dass es zu einer posttraumatischen Belastungsstörung (PTSD) kommt. Während der Pandemie durch die Coronaviruserkrankung 2019 (COVID-19) nahm die Belastung für die Angehörigen noch zu. In einer Studie von Zante et al. (2021) zeigten 90 % der Angehörigen Symptome einer PTSD [34].

Rund 70–78 % der Angehörigen sind von starker Angst betroffen

**Figure 1:**
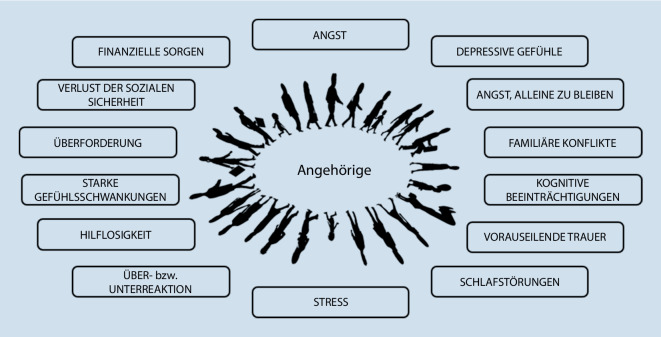

Speziell nach einem Aufenthalt auf der ICU wird von einem „family intensive care unit syndrome“ (FICUS; [26]) oder von der „postintensive care syndrome family“ (PICS-F) gesprochen. Das PICS‑F kann Monate oder Jahre andauern und mit sehr unterschiedlichen Auswirkungen, vorwiegend mit psychischen Einschränkungen, die Lebensqualität und Lebenssituation nachteilig beeinflussen [3, 23]. In Abb. 1 findet sich eine Übersicht über häufige Belastungen, die Angehörige während und nach dem ICU-Aufenthalt erleben.

Angehörige nehmen eine komplexe und vielschichtige Rolle ein. Sie sind mitbetroffen, vulnerabel und benötigen Unterstützung. Zusätzlich sind sie Stellvertreter*innen der Patient*innen und treten für deren Wünsche, Bedürfnisse und Vorlieben ein. Weiters werden sie in therapeutische, mitunter lebensentscheidende Entscheidungen eingebunden, überdies als Cotherapeut*innen um Mithilfe gebeten, z. B. bei der Reorientierung zur Delirprävention, Empowerment und Rehabilitation. Angehörige gelten damit als Teil des Behandlungs- und Betreuungsteams und sind für das Wohlbefinden und die Nachsorge der kritisch Kranken essenziell [6]. Eine Verschlechterung der psychischen und physischen Situation der Angehörigen hat damit auch immer eine Auswirkung auf die kritisch Kranken.

Angehörige sind Teil des Betreuungsteams und für das Wohlbefinden der kritisch Kranken essenziell

**Figure 2:**
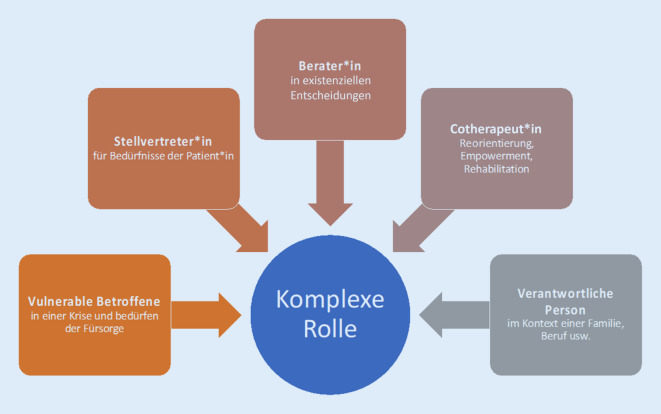

Schlussendlich sind sie noch selbständige Personen, die verantwortlich für Familie, Kinder, Finanzen und weiteres sind. Diese komplexe Rolle der Angehörigen wird in Abb. 2 verdeutlicht.

Die Anwesenheit und die Einbindung von Angehörigen in die Betreuung ist für Patient*innen extrem wichtig, weil die Angehörigen neben der emotionalen Begleitung wichtige Informationen zu Vorerkrankungen oder den individuellen Bedürfnissen der Patient*innen zur Verfügung stellen können [1]. Auch für die Angehörigen selbst ist die Anwesenheit und die Mithilfe auf der ICU wichtig [6, 20].

## Zielsetzung

Das Ziel dieses Beitrags ist es, eine Übersicht über die aktuellen Entwicklungen hinsichtlich der Angehörigenbegleitung, unter besonderer Berücksichtigung von COVID-19 und virtueller Angebote, darzustellen. Als grundlegende Orientierung dient die Leitlinie der Society of Critical Care Medicine (SCCM) zur familienorientierten Versorgung in der Intensivmedizin [6]. Da die Leitlinie aufgrund der schnell zunehmenden Forschungserkenntnisse zurzeit überarbeitet wird und die Pandemie viele Rahmenbedingungen wesentlich beeinflusst hat, werden hier die aktuellen Entwicklungen und Empfehlungen als narratives Review dargestellt.

## Ergebnisse

Die in Folge dargestellten Maßnahmen sind eine Zusammenfassung der übersetzten Leitlinien von Davidson et al. (2017; [6]). Die Autor*innen haben diese Liste um weitere Maßnahmen (unter anderem bezogen auf COVID-19 sowie virtuelle Angebote) ergänzt [17] und aktualisiert. Die wesentlichen Entwicklungen und Maßnahmen lassen sich 6 Bereichen zuordnen:

1. *Anwesenheit der Angehörigen:*
  offene oder flexible Besuchszeiten, Präsenz bei Visiten und Interventionen und Offenheit für die Besuche von Angehörigen unter 18 Jahren;
2. Einbindung in die Betreuung:
  routinemäßige Angehörigenedukation (z. B. Selbstmanagement, Selbstfürsorge), multidisziplinäres Führen von ICU-Tagebüchern und deren routinemäßiger Einsatz;
3. *Kommunikation, Information sowie Onlineangebote:*
  Informationsmaterialien (papierbasiert, online) für medizinische Laien mit Inhalten rund um die ICU, Kommunikationspläne sowie Standard Operating Procedures (SOP) bei Besuchseinschränkungen durch z. B. COVID-19 oder bei großen Entfernungen;
4. *Multidisziplinäre Zusammenarbeit:*
  Zusammenarbeit von Psycholog*innen, Sozialarbeiter*innen und Rehabilitationsspezialist*innen usw. im interdisziplinären Team
5. *Aufgaben der Organisationsleitung:*
  Implementierung von Qualitäts- und Risikomanagementmaßnahmen im Hinblick auf Angehörigenbegleitung, Förderung von (wissenschaftlichen) Projekten hinsichtlich der Einbindung von Angehörigen oder der neuen Rollen wie die von Familienpflegenden (Family Health Nurses);
6. *Follow-up-Betreuung:*
  ambulante Sprechstunden und Telefonate (multidisziplinär) nach einem Aufenthalt auf der ICU.

In Tab. 1 findet sich eine Zusammenfassung der einzelnen Maßnahmen zur Angehörigenbegleitung dargestellt in den sechs Bereichen.

**Table 1:** 

Maßnahme	Beschreibung
1. Anwesenheit der Angehörigen	Offene oder flexible Besuchszeiten
	Professionelle Begleitung von Angehörigen unter 18 Jahren angepasst an die individuelle Entwicklung
	Nach Möglichkeit und auf Wunsch Teilnahme der Angehörige an interdisziplinären Teamrunden und Visiten
	Nach Möglichkeit und auf Wunsch Anwesenheit bei Wiederbelebungsmaßnahmen
	Auf Wunsch sollten Patient*innen mit ihren Angehörigen die ICU z. B. vor oder nach einem großen Eingriff vor Ort oder virtuell besuchen dürfen
2. Proaktive Einbindung der Angehörigen	Angehörigen wird das Angebot gemacht zu lernen, wie sie bei der Pflege oder Therapie helfen können
	Angehörigenedukation erfolgt routinemäßig (z. B. Selbstmanagement, Selbstfürsorge)
	Eine Peer-to-Peer-Unterstützung wird implementiert (Erwachsene und Kinder)
	ICU-Tagebücher werden multidisziplinär geführt und routinemäßig eingesetzt
	Das Einbringen von persönlichen Gegenständen, wie Bildern, Duft, Musik etc., wird auf Wunsch ermöglicht
	Instrumente und Methoden (z. B. ethische Beratung) zur Entscheidungsunterstützung für Angehörige sind vorhanden
3. Strukturierte Kommunikation und Information sowie Onlineangebote	Routinemäßige interdisziplinäre Angehörigenkonferenzen werden genutzt
	Informationsmaterialien für medizinische Laien mit Inhalten rund um die ICU werden zur Verfügung gestellt
	Es wird eine Onlineunterstützung/-information für medizinische Laien angeboten
	Mitarbeiter*innen erhalten angehörigenzentrierte Kommunikationstrainings (Kommunikation in Krisen)
	Es gibt Kommunikationspläne und SOP bei Besuchseinschränkungen durch z. B. COVID-19 oder bei großen geographischen Entfernungen
	Bei fremdsprachigen Angehörigen werden Übersetzer*innen hinzugezogen
	Besonders bei Patient*innen, die eine schlechte Prognose haben, wird ein evidenzbasierter Kommunikationsansatz (Art der Kommunikation) angewendet, um die Kommunikation zwischen ICU-Team und Angehörigen zu erleichtern
	Maßnahmen in der Trauerbegleitung, wie Care-Teams und Nachbesprechungen, werden angeboten
4. Multidisziplinäre Zusammenarbeit	Proaktive Palliativberatungskonsultationen werden durchgeführt
	Ethikkonsultationen werden bei terminal Kranken durchgeführt
	Anwesenheit von Psycholog*innen bei Bedarf wird forciert
	Sozialarbeiter*innen werden in das interdisziplinäre Team aufgenommen, um z. B. an Angehörigentreffen teilzunehmen
	Angehörigennavigator*innen (z. B. Bezugspflege, Familienpflege oder Kommunikationsvermittler*innen) werden Familien während des gesamten ICU-Aufenthalts zugewiesen
	Familien soll spirituelle Unterstützung angeboten werden
	Rehabilitationsspezialist*innen der unterschiedlichen Berufsgruppen werden eingebunden oder vermittelt
5. Aufgaben der Organisationsleitung	Krankenhäuser implementieren Richtlinien zur Förderung der angehörigenorientierten Betreuung und Pflege auf der ICU, um die Zufriedenheit und die ICU-Erfahrung zu verbessern
	Implementation von Protokollen und SOP, die eine angemessene und standardisierte Verwendung von Sedierung und Analgesie während der letzten Lebensphase sicherstellen
	Es werden vom Krankenhausmanagement notwendige Kommunikationstools, wie z. B. Smartphones oder Tablets, zur Verfügung gestellt
	Pflegepersonen werden in die Entscheidungsfindung über die Ziele der Betreuung/Pflege einbezogen und werden geschult
	Die Lärmbelastung soll reduziert werden; ruhige/private Räume für Angehörige sollen zur Verfügung gestellt werden
	Es sollen Aufenthaltsbereiche mit der Möglichkeit einer Verpflegung und Schlafmöglichkeiten für Angehörige zur Verfügung gestellt werden
	Implementierung von Qualitäts- und Risikomanagementmaßnahmen hinsichtlich der Angehörigenbegleitung
	Förderung von (wissenschaftlichen) Projekten hinsichtlich der Unterstützung und Einbindung von Angehörigen
	Unterstützung von neuen Rollen in den verschiedenen Berufsgruppen wie z. B. Familienpflegende (Family Health Nurse) oder die Gemeindeschwester (Community Nurse)
6. Follow-up-Angebote	Multidisziplinäre Follow-up-Angebote, wie Sprechstunden, Telefonate, nach einem Aufenthalt auf der ICU, Follow-up-Kliniken für Patient*innen und Angehörige sowie Peer-Support werden strukturiert implementiert

*COVID-19 *Coronaviruserkrankung 2019, *ICU* Intensivstation, *SOP* Standard Operating Procedure

## Herausforderung durch Besuchsrestriktionen aufgrund von COVID-19

Bei der COVID-19-Pandemie kam es gerade zu Beginn zu massiven Besuchseinschränkungen oder gar Besuchsverboten. Oftmals durften selbst Sterbende nicht mehr besucht werden. Boulton et al. (2021) berichtet, dass in dieser Zeit rund 22 % der ICU keine Besuche gestatteten und rund 53 % nur am Ende des Lebens [5]. Viele bereits implementierte Maßnahmen in der Angehörigenbetreuung wurden unterbrochen oder adaptiert. So mussten die gesamte Beziehungsarbeit und Informationsweitergabe mit und an die Angehörigen über veränderte Kanäle und Konzepte erfolgen. Das stellte auch für die Gesundheitsfachpersonen eine sehr hohe Belastung dar [19].

Oftmals durften selbst Sterbende nicht mehr besucht werden

Die Deutsche Interdisziplinäre Vereinigung für Intensiv- und Notfallmedizin (DIVI) hat bezüglich der Besuchsregelung von Angehörigen während Pandemien ein Positionspapier verfasst [32]. Ziel dabei ist es, täglich alle stationären Patient*innen zu evaluieren und abzuschätzen, ob ein Besuch zu mehr Vor- als Nachteile führen könnte. Dabei müssen Patient*innen und Angehörigen in diesen Prozess miteingebunden werden und darüber informiert werden, welche Risiken im individuellen Fall existieren.

## Onlineangebote

Verständliche Informationen zu erhalten, ist ein wichtiges Grundbedürfnis der Angehörigen [13]. Laut Fergé et al. (2018) berichten rund 80 % der Angehörigen von kritisch Kranken über ein Wissensdefizit [10]. Dies ist vor dem Hintergrund der Komplexität der Erkrankung und Behandlung, der fehlenden Zeitressourcen der Mitarbeiter*innen [28], der Besuchseinschränkungen und der individuell ausgeprägten Gesundheitskompetenz verbunden mit einer großen Unsicherheit hinsichtlich mancher Prognosen auch nicht verwunderlich.

Rund 80 % der Angehörigen von kritisch Kranken berichten über ein Wissensdefizit

Etwa die Hälfte der Angehörigen von kritisch Kranken sucht nach Informationen im Internet [4, 12]. Dabei stoßen sie zumeist auf Informationen, die für medizinische Laien nicht geeignet sind, weil die Informationen z. B. in medizinischer Fachsprache oder auf Englisch verfasst wurden. Oftmals stammen die Informationen auch aus Foren, in denen andere medizinische Laien versuchen, Fragen zu beantworten oder Sachverhalte zu erklären. Zusätzlich gibt es auch Informationen seitens der Industrie, die möglicherweise durch Interessenskonflikte beeinflusst sind. Die Gesundheitskompetenz der Bevölkerung, also die Fähigkeit der Menschen, relevante Gesundheitsinformationen zu finden, zu verstehen, zu beurteilen und anzuwenden, ist individuell sehr unterschiedlich ausgeprägt [12, 31] und daher besteht auch die Gefahr, dass Informationen missverstanden werden.

Das im Jahr 2016 initiierte ICU-Families-Projekt hat sich zum Ziel gesetzt, kritisch Kranke und ihre Angehörigen im Raum Deutschland, Österreich, Schweiz (DACH) zu informieren und zu unterstützen. Dazu wurde zuerst der Informationsbedarf der Angehörigen erhoben. Hoffmann et al. (2018) berichtet dabei von divergierenden Ergebnissen zwischen den tatsächlichen Bedürfnissen der Angehörigen und den vermuteten der Expert*innen. Die in Folge entwickelte Webseite mit Informationen rund um die ICU wurde dann im Rahmen einer randomisierten kontrollierten Studie untersucht. Die Ergebnisse werden derzeit für eine Publikation vorbereitet.

Das ICU-Families-Projekt informiert und unterstützt kritisch Kranke und ihre Angehörigen

**Figure 3:**
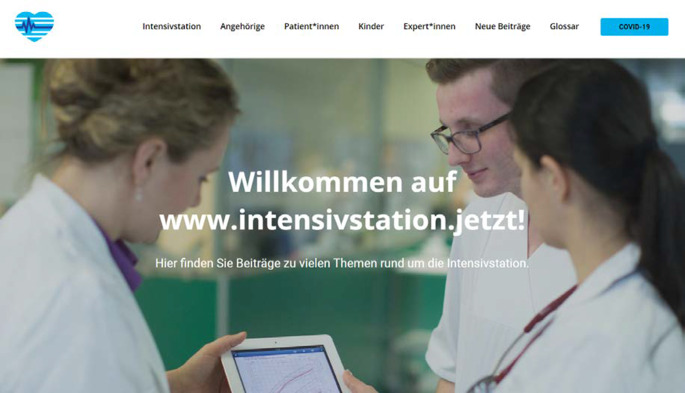

Aufgrund der COVID-19-Pandemie wurde die Webseite vorzeitig überarbeitet und steht nun seit dem Frühjahr 2021 unter www.intensivstation.jetzt für Angehörige, Patient*innen und interessierte Expert*innen zur Verfügung. Die Webseite wird ehrenamtlich von einem internationalen und multidisziplinären Team aus dem DACH-Raum betreut und kann mit wenigen einfachen Schritten auf jeder ICU implementiert werden. Eine Anleitung dazu findet sich direkt auf der Webseite (https://www.intensivstation.jetzt/expertinnen/, siehe dazu Abb. 3 und 4).

**Figure 4:**
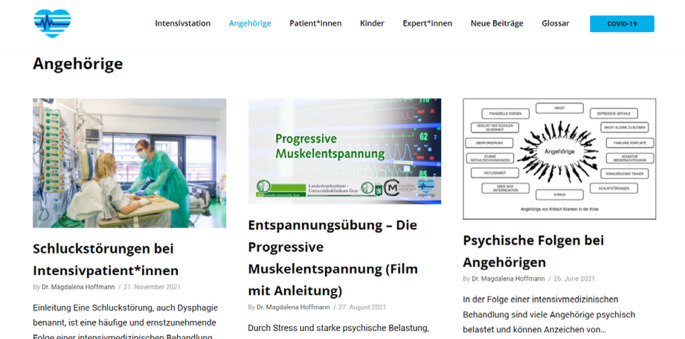

Im weiteren Projektverlauf werden auf der Webseite laufend neue und relevante Informationen rund um die ICU ergänzt, sodass allgemeine Themen, Abläufe und Herausforderungen rund um die ICU für medizinische Laien gut und einfach erklärt sind. Die Webseite soll die Kommunikation mit den Mitarbeiter*innen der ICU ergänzen und steht 24 h zur Verfügung.

### Follow-up-Angebote

Follow-up-Angebote sind mono- oder multiprofessionelle Maßnahmen für Patient*innen und ihre Angehörigen. Diese Angebote kommen nach einer kritischen Erkrankung und deren Therapie auf der ICU zur Anwendung und leisten einen Beitrag zur physischen und psychischen Genesung der Menschen. Im angloamerikanischen Raum sowie in Skandinavien sind Angebote wie Follow-up-Sprechstunden, Telefonate nach einem Aufenthalt auf der ICU, Follow-up-Kliniken für Patient*innen und Angehörige, Peer-Support und Intensivtagebücher bereits häufiger implementiert. Über die Wirksamkeit dieser Angebote ist bisher jedoch noch wenig bekannt [30]. Zudem werden die bereits genannten Unterstützungsangebote im deutschsprachigen Raum noch sehr selten umgesetzt. Belastungsstörungen bleiben so weitgehend unerkannt/unbehandelt und können sich nicht nur gravierend auf das Leben der Betroffenen, sondern auch auf die Gesellschaft auswirken.

Follow-up-Angebote leisten einen Beitrag zur physischen und psychischen Genesung

Besuche im Krankenhaus, Folgebesuche auf der ICU oder Telefonate sind weitere unterstützende Angebote für Patient*innen und Angehörige [30] nach dem ICU-Aufenthalt. Bei diesen Angeboten werden beispielsweise alle ehemals kranken Personen, die über eine bestimmte Anzahl von Tagen auf der ICU waren oder unter einer bestimmten Erkrankung litten, mit ihren Angehörigen zu einem Gespräch eingeladen. Im multidisziplinär geführten Gespräch werden aktuelle medizinische oder therapeutische Fragen geklärt und Fragen zum ICU-Aufenthalt beantwortet. Das können

- die Herausforderungen mit der fehlenden körperlichen Kraft,
- die nächsten wichtigen Schritte wie medizinische oder therapeutische Konsultationen oder
- die Besprechung von psychischen Symptomen

sein. Hier stehen die Erlebnisse, Erinnerungen und Träume der Kranken während des Intensivaufenthalts im Mittelpunkt. Dabei findet zudem ein erster Vergleich der Erinnerungen mit den tatsächlichen Gegebenheiten statt. In dieses Gespräch werden häufig validierte Assessmentinstrumente eingebaut, so z. B. die Erfassung der aktuellen Lebensqualität. Anzeichen von psychischen Belastungen, wie Flashbacks, PTSD und Schlafstörungen, können so ermittelt werden. Diese Gespräche und Besuche haben zum Ziel, die Betroffenen in der Verarbeitung der erlebten Vergangenheit zu unterstützen. Die Besprechung von zukünftigen Zielen und Maßnahmen, die helfen können, den Gesundheitszustand zu verbessern, ergänzen die Angebote.

In Peer-Support-Gruppen unterstützen sich Betroffene gegenseitig

Ehemalige Patient*innen, Angehörige und die Fachliteratur weisen immer häufiger auf den Nutzen von Peer-Groups hin [15]. So berichten Mikkelsen et al. (2016), wie sich Betroffene in Peer-Support-Gruppen (Selbsthilfegruppen) gegenseitig unterstützt haben [25]. Das Behandlungsteam der ICU organisierte die Treffen für ehemalige Patient*innen und deren Angehörige, damit diese sich austauschen konnten. Gespräche über den Aufenthalt auf der ICU, den Genesungsverlauf und aktuelle Probleme konnten dabei Hoffnung und Zuversicht vermitteln. Die Teilnehmenden erhielten Informationen oder erlernten Möglichkeiten, wie sie die Gesundheit und das Wohlbefinden fördern, das Selbstmanagement unterstützen und die psychische Widerstandsfähigkeit (Resilienz) stärken können [15].

Das Intensivtagebuch findet im deutschsprachigen Raum immer mehr Verbreitung

Das Intensivtagebuch findet im deutschsprachigen Raum immer mehr Verbreitung (siehe www.intensivtagebuch.de). Es dient dazu, Erinnerungslücken zu füllen, die Patient*innen im Zusammenhang mit dem Aufenthalt auf der ICU haben. Erlebnisse werden verständlicher und ergeben einen Sinn [9], lassen sich bewältigen und verarbeiten. Pflegefachpersonal und das multiprofessionelle Team, beispielsweise Ärzt*innen oder Physiotherapeut*innen, schreiben die Einträge. In der Praxis übernehmen insbesondere die Pflegefachpersonen das Führen der Tagebücher. Bisherige Studien diskutieren den Nutzen von Tagebüchern kontrovers [11, 33], jedoch wird das Tagebuch von Patient*innen, Angehörigen und Fachpersonen als sehr hilfreich erlebt [8, 24].

Die Empfehlungen der SCCM zur familienorientierten Versorgung auf der ICU bieten eine gute Übersicht zu den bereits angeführten Maßnahmen. Wichtige Ergänzungen sind Onlineangebote, die einfach zu implementieren sind. Neben Informationen über Websites, Follow-up-Angebote und Intensivtagebücher gibt es erste Ideen für eine kognitive Verhaltenstherapie per Smartphone (z. B. Apps) zur Vermeidung einer PTSD [27] oder digitale ICU-Tagebücher [14]. Das sind erfolgversprechende Ideen, die die individuelle Belastung der Angehörigen sowie möglicherweise den Einsatz von Psychopharmaka zur Bekämpfung von Symptomen einer PTSD vermindern könnten.

Die Umsetzung der einzelnen Angebote für Angehörige muss in enger Abstimmung zwischen dem Management der Organisation und den Mitarbeiter*innen sowie gegebenenfalls unter Begleitung von internen oder externen Expert*innen erfolgen. Es gilt, die Maßnahmen immer wieder zu evaluieren und gezielt an die Bedürfnisse der Angehörigen nach Evaluierung anzupassen.

Einfach zu implementierende Onlineangebote sind wichtige Ergänzungen

Anzumerken ist, dass sich die Finanzierung der Gesundheitssysteme in den einzelnen Ländern stark unterscheidet. Das heißt mitunter auch, dass Maßnahmen und Angebote wie z. B. Follow-up Angebote nicht durch die Gesundheitseinrichtungen – an denen die ICU angesiedelt sind – verrechnet werden können. Das stellt eine reelle Hürde bei der Finanzierung solcher Angebote dar.

## Limitationen

Bei den hier dargestellten Maßnahmen und Projekten handelt es sich um eine von den Autor*innen ausgewählte Übersicht, die laufend ergänzt wird. Daher kann diese Übersicht nicht als umfassend oder abschließend betrachtet werden. Zusätzlich ist anzumerken, dass die hier angeführten Maßnahmen keiner individuellen Betrachtung hinsichtlich ihrer Evidenz bezüglich einer Wirksamkeit auf bestimmte Parameter wie z. B. Angehörigenzufriedenheit oder Reduktion von Stresssymptomen unterzogen wurden.

## Schlussfolgerungen

Angehörige befinden sich in einer komplexen Rolle: Sie sind Angehörige und gleichzeitig Teil des Behandlungsteams. Zusätzlich zeigt die COVID-19-Pandemie, die häufig eine Reduktion der Personalkapazitäten sowie Besuchseinschränkungen mit sich bringt, wie wichtig ergänzende Maßnahmen sind, um die betroffenen Angehörigen und die kritisch Kranken während und nach dem ICU-Aufenthalt zu unterstützen. Die Umsetzung der genannten Angebote darf dabei nicht nur von der Motivation und Kraft einzelner Mitarbeiter*innen abhängig sein.

Vielmehr bedarf es hier eines Anerkennens dieser Bedürfnisse und der Herausforderungen der Angehörigen und kritisch Kranken durch Entscheidungsträger*innen sowie der Bereitstellung von Ressourcen, damit präventive Maßnahmen und Konzepte implementiert werden können. Die professionelle Begleitung der Angehörigen ist nicht nur ethisches Gebot, sondern auch ein Beweis der Professionalität der Mitarbeiter*innen und des Gesundheitssystems.

## Fazit für die Praxis

- Angehörige von kritisch Kranken befinden sich in einer komplexen Rolle und bedürfen der Fürsorge, Begleitung und Edukation.
- Frühzeitig angewendete Maßnahmen in der Angehörigenbegleitung können möglicherweise eine Verschlechterung der Lebensqualität der Angehörigen verhindern, sodass diese später besser in der Lage sind, sich um sich selbst und um die kritisch Kranken nach Entlassung aus der Intensivstation zu kümmern.
- Es bedarf einer gemeinsamen Entscheidung der Mitarbeiter*innen und des Managements, welche Maßnahmen für Angehörige auf der Intensivstation umgesetzt werden können.
- Die Umsetzung der geplanten Maßnahmen sollte, unter entsprechender Bereitstellung der notwendigen Ressourcen, regelmäßig evaluiert werden.
